# Citrus Huanglongbing correlated with incidence of *Diaphorina citri* carrying *Candidatus* Liberibacter asiaticus and citrus phyllosphere microbiome

**DOI:** 10.3389/fpls.2022.964193

**Published:** 2022-11-18

**Authors:** Yang Hu, Youqing Meng, Liangjin Yao, Enguo Wang, Tao Tang, Yunsheng Wang, Liangying Dai, Mingping Zhao, Hong-en Zhang, Xiaoyan Fan, Luyun Luo, Wei Xiang, Zhuo Zhang

**Affiliations:** ^1^ Department of Tree Breeding, Zhejiang Academy of Forestry, Hangzhou, China; ^2^ Zhejiang Provincial General Station of Plant Protection, Quarantine and Pesticide Management, Hangzhou, China; ^3^ Department of Plant Protection, Zhejiang Linhai Agricultural Technology Extension Center, Taizhou, China; ^4^ Hunan Plant Protection Institute, Hunan Academy of Agricultural Science, Changsha, China; ^5^ College of Plant Protection, Hunan Agricultural University, Changsha, China; ^6^ Plant Protection and Quarantine Station, Bureau of Agriculture and Rural Affairs of Jianghua Yao Autonomous County, Yongzhou, China; ^7^ School of Mathematical, Nankai University, Tianjin, China; ^8^ Institute of Environmental Biotechnology, Graz University of Technology, Graz, Austria; ^9^ School of Advanced Agriculture and Bioengineering, Yangtze Normal University, Chongqing, China; ^10^ Hunan Crop Research Institute, Hunan Academy of Agricultural Science, Changsha, China

**Keywords:** citrus huanglongbing, *Candidatus* Liberibacter asiaticus, *Diaphorina* citri, phyllosphere microbiome, pathogen detection and monitoring

## Abstract

In China, citrus Huanglongbing (HLB) disease is caused by the *Candidatus* Liberibacter asiaticus bacterium, which is carried by the Asian citrus psyllid *Diaphorina citri* Kuwayama. It was hypothesized that the epidemic of the HLB may related with the rate of bacterium presence in the insect vector and bacterium content in plant tissues, as well as the phyllosphere microbe communities changes. This study systematically analyzed the presence or absence of *Ca.* L. asiaticus in citrus tree leaves and in the insect vector *D. citri* over a 6-year period using real-time PCR. In addition, changes in the number of bacteria carried by *D. citri* over 12 months were quantified, as well as the relationship between the proportion of *D. citri* carrying *Ca.* L. asiaticus and the proportion of plants infected with *Ca.* L. asiaticus were analyzed. Results showed that the proportion of *D. citri* carrying bacteria was stable and relatively low from January to September. The bacteria in citrus leaves relatively low in spring and summer, then peaked in December. The proportion of *D. citri* carrying bacteria gradually declined from 2014 to 2019. The proportion of *D. citri* carrying *Ca.* L. asiaticus showed a significant positive correlation with the proportion of diseased citrus. The phyllosphere bacterial and fungal communities on the healthy citrus leaf were significantly different with the disease leaf in April and December. Pathogenic invasions change the citrus phyllosphere microbial community structure. It could be summarized that citrus Huanglongbing correlated with incidence of *Diaphorina citri* carrying *Candidatus* Liberibacter asiaticus and citrus phyllosphere microbiome.

## Highlights

(1) We monitored citrus Huanglongbing (HLB) for 6 years to obtain enough samples and accumulate long-term continuous data to analyze the spread and prevalence of Huanglongbing in Zhejiang Province, China.
(2) The number of pathogenic bacteria in citrus leaves and the rate of insect vector *Diaphorina citri* carrying bacterium was lowest in spring and highest in December.
(3) Phyllosphere microorganisms of citrus are correlated with HLB.

## Introduction

Citrus Huanglongbing (HLB) disease has been reported over 50 countries around the world, which causes serious damage to the citrus industry (Faghihi et al., 2009; Gottwald, 2010; Lopes et al., 2010). HLB is caused by a group of bacteria called *Candidatus* Liberibacter that inhabit in the phloem of citrus trees. Three species of HLB-causing bacterium have been reported: *Candidatus* Liberibacter africanus, *Candidatus* Liberibacter asiaticus and *Candidatus* Liberibacter americanus. The bacteria can infect different tissues of host plant once invading, and then affect plant growth and development, such as causing metabolism disorders, leaves yellowing, fruits deformity, and roots rot (Pustika et al., 2008; Koh et al., 2012; Etxeberria et al., 2009; Johnson et al., 2014). Infected citrus trees will be significantly shortened profitable lifetimes and lower yields (Gottwald, 2010).

In plant pathological systems, many parasites infect plants and increase their prevalence by host vectors. HLB is transmitted by insect vectors feeding on the phloem of citrus foliage. The HLB causing by africanus species is transmitted by the African citrus psyllid *Trioza erytreae*. Meanwhile, the HLB causing by the asiaticus and americanus species is transmitted by the Asian citrus psyllid *Diaphorina citri* Kuwayama (Hemiptera: Liviidae) (Grafton-Cardwell et al., 2013). Studies have shown that HLB disease in China is caused by *Ca.* L. asiaticus, which is associated with the vector *D. citri* (Hall et al., 2013). Suitable growth area of the bacteria and the insect hosts has been expanded with the rising winter temperatures in recent decades due to global warming (Wang et al., 2020). Bacteria are acquired by *D. citri* when they feed on the infected plant, after that, bacteria will proliferate in *D. citri* and maintain throughout the life history of the adult psyllid (Aubert and Quilici, 1984; Tabachnick, 2015; Luo et al., 2016). Citrus HLB is optimal and limitted by temperature conditions (Narouei-Khandan et al., 2015). However, there is still no long-term quantitative monitoring of bacterial content levels in different hosts, and lack of understanding of this aspect.

The population dynamics of insects are closely related to the growth rhythm, desirable food intake and nutritional quality of host plants (Wallner, 1987). The phenological characteristics of host plants will affect the growth of insects, leading to genetic variation among insect individuals and genetic differentiation among insect populations (Knolhoff and Heckel, 2014). Although tremendous progress has been made in understanding the ecological and evolutionary underpinnings of the Liberibacter disease pyramid, little is known about the quantitative relationship between these factors in the pyramid.

The phyllosphere (aboveground part of terrestrial plants) is an important niche of the plant, inhabited by diverse microbes which are collectively called the phyllosphere microbiome (Vorholt, 2012). The phyllosphere microorganisms could influence host plant by affecting nutrient acquisition, promoting host stress tolerance, altering plant hormones, and mediating plant pathogen interactions (Stone et al., 2018). The phyllosphere microbiomes were found to differ between infected and uninfected citrus leaves by melanose pathogen, and part of the phyllosphere microbiome shift could positively affect plant performance against pathogen invasion (Li et al., 2022).

**Figure 1:**
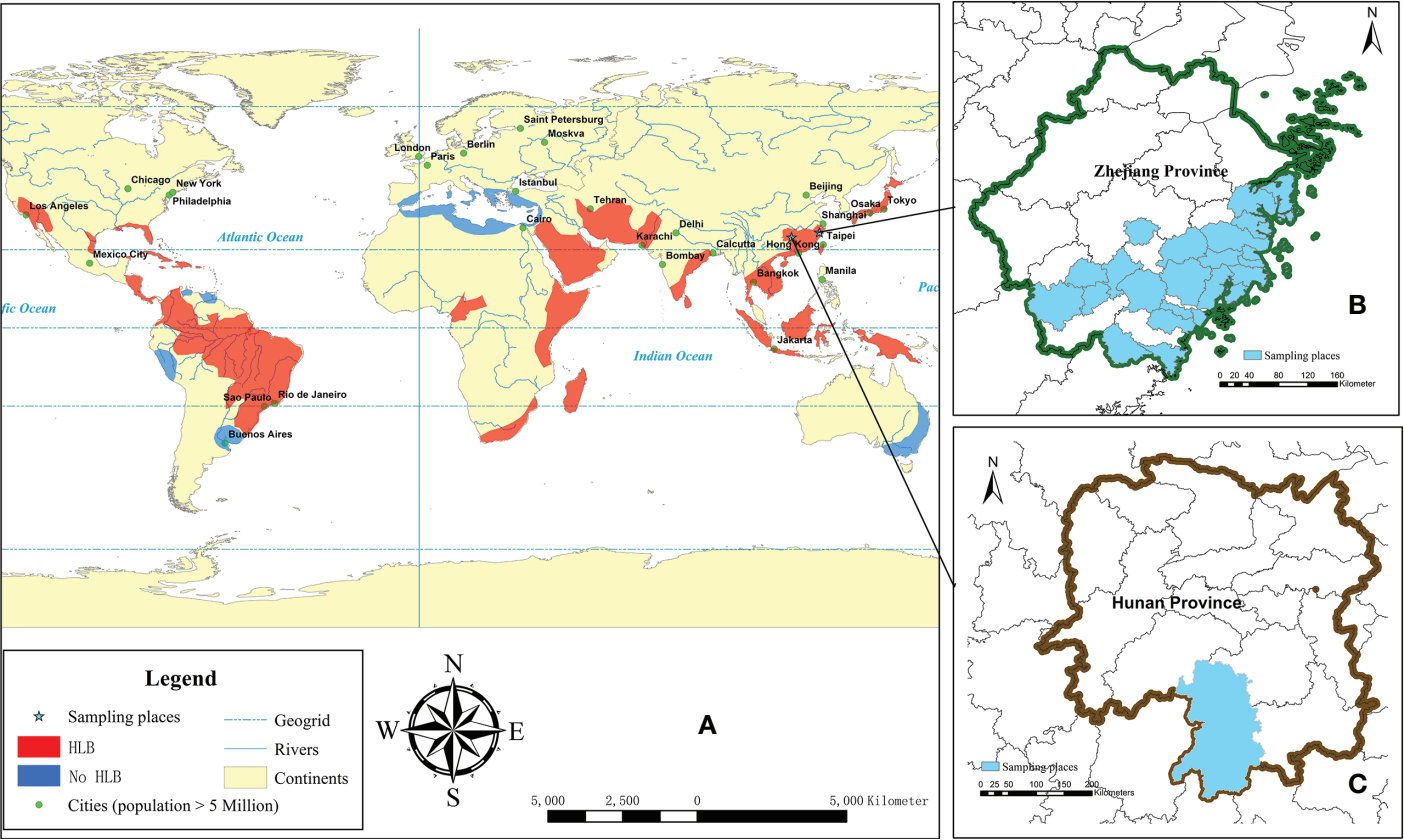
Illustration of the major citrus growing areas (orange and blue), HLB happened areas (orange) adapted from Dala-Paula et al. (2018) and Liu et al. (2012) **(A)**, and the sampling areas in Zhejiang **(B)** and Hunan **(C)** province.

In this study, we hypothesizes that the epidemic of the HLB may related with the rate of bacterium presence in the insect vectors and bacterium content in plant tissues, as well as the phyllosphere microbe communities. *Citrus unshiu* orchards in different regions of Zhejiang and Hunan Province were systematically analyzed to determine the level of threat from HLB disease (**Figure 1**). The number of *D. citri* carrying *Ca.* L. asiaticus over time was quantified. The phyllosphere microbiomes of healthy citrus leaves and HLB diseased leaves were investigated in April and December to understand the correlation among phyllosphere microbial community structure, HLB disease and seasonal effects. The aim of this study is to determine the influence of the bacteria carrier rate of *D. citri* in citrus orchards at different growth stages, in order to provide a theoretical basis for future exploring the ecological regulation and comprehensive control of *D. citri* and *Ca.* L. asiaticus.

## Materials and methods

### Orchards location and sample collection

For monitoring the *D. citri* carring *Ca.* L. asiaticus over a six-year period from 2014 to 2019, random sites from locations in different counties in the south of Zhejiang Province were chosen for sample collecting (**Figure 1**, **Table 1**).

**Table 1:** Proportion of *Diaphorina citri* carrying *Candidatus Liberibacter* asiaticus bacteria in orchards in southern counties in Zhejiang Province from 2014 to 2019.

Years	locations and No.of Samples took	No.of Samples detected with HLB
2014	Cangnan (10), Ruian (10), Ouhai (10), Yongjia (10), Yueqing (10), Longquan (10), Yunhe (10), Qingtian(20), Songyang(10), Liandu (10), Yuhuan (10),Wenling(10), Luqiao (10), Jiaojiang (10), Linhai (10), Shanmeng (10), Xianju (10), Ninghai (10), Xiangshan (10), Yongkang (20)	Cangnan (6), Ruian (7), Ouhai (3), Yongjia (3), Yueqing (6), Longquan (4), Yunhe (5), Qingtian (4), Songyang (9), Liandu (8), Yuhuan (10), Wenling (4), Luqiao (4), Jiaojiang (4), Linhai (1), Shanmeng (3), Xianju (0), Ninghai (3), Xiangshan (1), Yongkang (4)
2015	Wenling (30), Liandu (30), Ninghai (12), Huangyan (10)	Wenling (13), Liandu (11), Ninghai (0), Huangyan (3)
2016	Yuhuang (20), Wenling (20), Luqiao (30), Jiaojiang (30), Linhai (10), Shanmeng (14)	Yuhuang (10), Wenling (10), Luqiao (10), Jiaojiang (10), Linhai (10), Shanmeng (10),
2017	Yongjia (24), Yueqing (60), Wenling (30), Liandu (30)	Yongjia (3), Yueqing (18), Wenling (10), Liandu(10)
2018	Ruian (30), Longwan (40), Yueqing (40), Wenling (10), Linhai (10)	Ruian (6) Longwan (10), Yueqing (10), Wenling (0), Linhai (8)
2019	Taishun (30), Wenling (20), Ruian (15) Longwan (63), Yueqing (61), Cangnan (97), Huangyan (17), Liandu (13), Qingtian (31)	Taishun (3), Wenling (15), Ruian (4) Longwan (34), Yueqing (7), Cangnan (23), Huangyan (1), Liandu (0), Qingtian (3)

All the *C. unshiu* trees monitored were over 12 years old and at the high yield stage. In each site, 60 psyllids were collected from one *C. unshiu* tree and stored into an individual 1.5-ml tubes, then frozen at –20°C for the future tests. DNA was extracted from 30 psyllids randomly selected from the 60 psyllids (the other 30 psyllids were stored for backup) and processed to detect and quantify *Ca.* L. asiaticus.

For monitoring the rate of *D. citri* and *C. unshiu* leaves carring *Ca.* L. asiaticus changes during the year, An orchards in Yueqing (Zhejiang Province), in which the disease incidence rate were over 90.0%, were sampled by using five-point sampling of the orchards, once a month for 12 months from May 2015 to April 2016. Five leaf samples were also collected from the same point where the insects collected to determine the bacterial content in the midvein. Approximately 45–70 insects were collected around the 20^th^ day of each month. Thirty insects of each month were processed to determine the presence of the bacterium.

To determine the rate of *D. citri* carring *Ca.* L. asiaticus in orchard with different disease incidence, psyllids were also collected by using five-point sampling of the orchards in the early August from 22 orchards in Huangyan, Yueqing, Wenling and Yuhuan (Zhejiang Province) in 2019 (**Table 2**).

**Table 2:** To study the relationship between the proportion of diseased plants and the proportion of *D. citri* carrying the bacterium, 22 different orchards located in Huangyan, Yuhuan, Wenling,and Yueqing were investigated.

tag	Location	Samled time	Investigated trees	% of deseased trees	Tested insects	The number of tested insects carrying bacterium	% oftested insects carrying bacterium
1	Huangyan	Aug-05	300	0	200	13	6.5
2	Huangyan	Aug-05	300	0	200	7	3.5
3	Huangyan	Aug-02	300	23.1	200	28	14
4	Huangyan	Aug-02	300	12.3	200	11	5.5
5	Huangyan	Aug-02	300	11.67	200	13	6.5
6	Huangyan	Aug-05	300	0	200	7	3.5
7	Huangyan	Aug-04	300	7	200	26	13
8	Huangyan	Aug-04	300	12.9	200	26	13
9	Huangyan	Aug-04	300	13.1	200	20	10
10	Huangyan	Aug-04	300	7	200	27	13.5
11	Huangyan	Aug-04	300	12.9	200	28	14
12	Huangyan	Aug-04	300	13.1	200	20	10
13	Yueqing	Aug-04	300	49.2	200	34	17
14	Yueqing	Aug-04	300	67	200	60	30
15	Yueqing	Aug-04	300	91.7	200	85	42.5
16	Yueqing	Aug-04	300	98.5	200	134	67
17	Yueqing	Aug-04	300	98	200	134	67
18	Wenling	Aug-04	300	64.8	200	74	37
19	Wenling	Aug-04	300	76.9	200	32	16
20	Yuhuan	Aug-02	200	100	200	156	78
21	Yuhuan	Aug-02	200	3.2	200	6	3
22	Yuhuan	Aug-02	300	1.5	200	3	1.5

Ten healthy and HLB leaves were collected by using five-point sampling within an area of 600 m^2^ for phyllosphere microbiome study at each sampling point in April (Spring) and December (Autumn), respectively, in Yongzhou (Hunan Province) in 2018. Ten leaf samples of each sampling point were mixed put into a sterile bag and refrigerated at -80°C. Every sterile bag contained 10 leaves which were cut into tiny pieces and the samples mixed into 4 treatments which are healthy Apirl (SK), diseased Apirl (SB), healthy December (AB) and diseased December (AK), before subsequent processing. The phyllosphere microorganisms were collected following the procedures of Xie et al. (2015). The microbes were filtered by a 0.22 μm filter microfiltration membrane using the air pump filtration, and then the samples were stored at -80°C for subsequent DNA extraction.

### Detection and quantification of *Ca.* L. asiaticus

The insects or leaves were crushed with a glass rod, and DNA was extracted using the CTAB extraction method. 400µL prechilled buffer (100 mmol/L Tris-HCl, 10 mmol/L EDTA, 700 mmol/L NaCl, pH 8.0) was added into the crushed powder, then added 500µL 65°C preheated buffer (2% CTAB, 50mmol/L Tris-HCl, 10 mmol/L EDTA, 800 mmol/L NaCl, pH 8.0). Samples were mixed and incubated at 65°C for 120 min, during which mixing the samples by inverting the tubes gently per 20 min. After that, add 450µL chloroform and mix by inverting the tubes and centrifuge 2 minutes at 12000r, the aqueous phase (above the white interface layer) to a clean microtube (then discard the rest), add 1 μL RNase (DNase-free) and incubate for 30 min at 37°C. Add 0.6 mL of isopropanol (2/3 of the recovered volume). Gently invert the microtube to be sure mixing is complete. Leave to precipitate for overnight at room temperature to precipitated the DNA, spin 15 min at 12000r at 4°C to pellet the DNA, remove the supernatant carefully, then wash the pellet once or twice with cold EtOH, spin 15 min at max speed, 4°C, remove supernatant and dry the pellet by leaving tube open, resuspend pellet in sterile H2O, store at -20°C (Winnepenninckx et al., 1993). The bacterium was quantified using RT-qPCR, The primers pair: 5’-CAAGG AAAGA GCGTA GAA-3’ and 5’-CCTCA AGATC GGGTA AAG-3’ were used. The PCR is carried by 25μL system with 2μL DNA and 0.3 μmol/L primers and 1X PCR master mix, on the iCycler™(Bio rad, USA) marchin with the program of 94°C, 5 minute, 40 cycles of 95°C, 5 second, 59°C, 15 second, and 72°C, 45 second, ended with 72°C, 7 minute. A standard curve was prepared and calculated using T vectors with DNA fragments of the target bacterial gene (382 bp) insertion. Copy numbers of T vectors were diluted to obtain copy numbers of 10, 10^2^, 10^3^, 10^4^, 10^5^, 10^6^ for a standard curve. The standard linear regression (Y=a+bX) of the log concentration of the target DNA copies(Y) versus the mean Ct value(X) were obtained (Li et al., 2008).

### DNA extraction, PCR amplification and pyrosequencing for phyllosphere microbiome

The total DNA was extracted from phyllosphere samples according to the manufacturer’s protocol using the MP FastDNA^®^ SPIN Kit for soil (MP Biochemicals, Solon, OH, USA). The PCR amplification were performed following Luo et al. (2019). The bacterial and fungi forward and reverse primers with a unique 12 nt barcode were included as the modification in the study, respectively. The bacterial and fungal ITS regions were amplified as previously described by Kong et al. (2019). PCR products were purified with an E.Z.N.A. ^®^ Gel Extraction Kit, pooled in equimolar amounts using Qubit (CA, USA). And mixed PCR products were sequenced (2×250 bp) on an Illumina MiSeq platform by ANNOROAD Gene Technology Co., Ltd. (Beijing, China) according to standard protocols.

Raw sequence data reads were processed with an in-house pipeline (http://mem.rcees.ac.cn:8080). In brief, a separate sample was generated according to a series of 12-bp barcodes and primers, and allowing for one mismatch. Paired-end reads (overlap > 30 bp) were combined by the FLASH program (Magoc & Salzberg, 2011). The combined sequences (Quality Score < 20) were filtered by Btrim program (Kong, 2011). Then the sequences with either an ambiguous base or the sequence length less than 200 bp were discarded. The UPARSE algorithms were used to detect and remove chimera sequences (Edgar, 2013). Low abundance OTUs (≤ 1 count) were eliminated from the OTU table. The microbial representative sequences for each OTU were assigned to taxonomic groups using the RDP Classifier database (Silva database 132 version) and UNITE database (Version 12.01.2017) (Abarenkov et al., 2010). The data were resampled randomly with the lowest sequence number (17,590 for bacteria and 27,286 sequences for fungi). The resampled OTU table was used for the subsequent analysis. In this study, all the microbial raw sequences were deposited in the SRA database short-read archive PRJNA844183.

### Statistical analysis

To plot the curve to fit the relationship between *D. citri* bacterial infection rate and the HLB incidence, the linear mixed model uses the lmer function performed in the “nlme” package, and all statistical analyses were performed in the R3.2.5 (R Core Team, 2016). The Chao1 and Inv_Simpson index were used to assess the difference of α diversity indices between healthy and diseased citrus phyllosphere samples. The weighted principal coordinate analysis (PCoA) based on UniFrac matrix and nonparametric permutational multivariate (PERMANOVA) based on Bray Curtis were used to assess the difference of microbial community structure between healthy and disease phyllosphere samples (Anderson, 2001; Caporaso et al., 2010).

## Results

### Quantitative detection of bacteria in the insect vector *D. citri*

**Figure 2:**
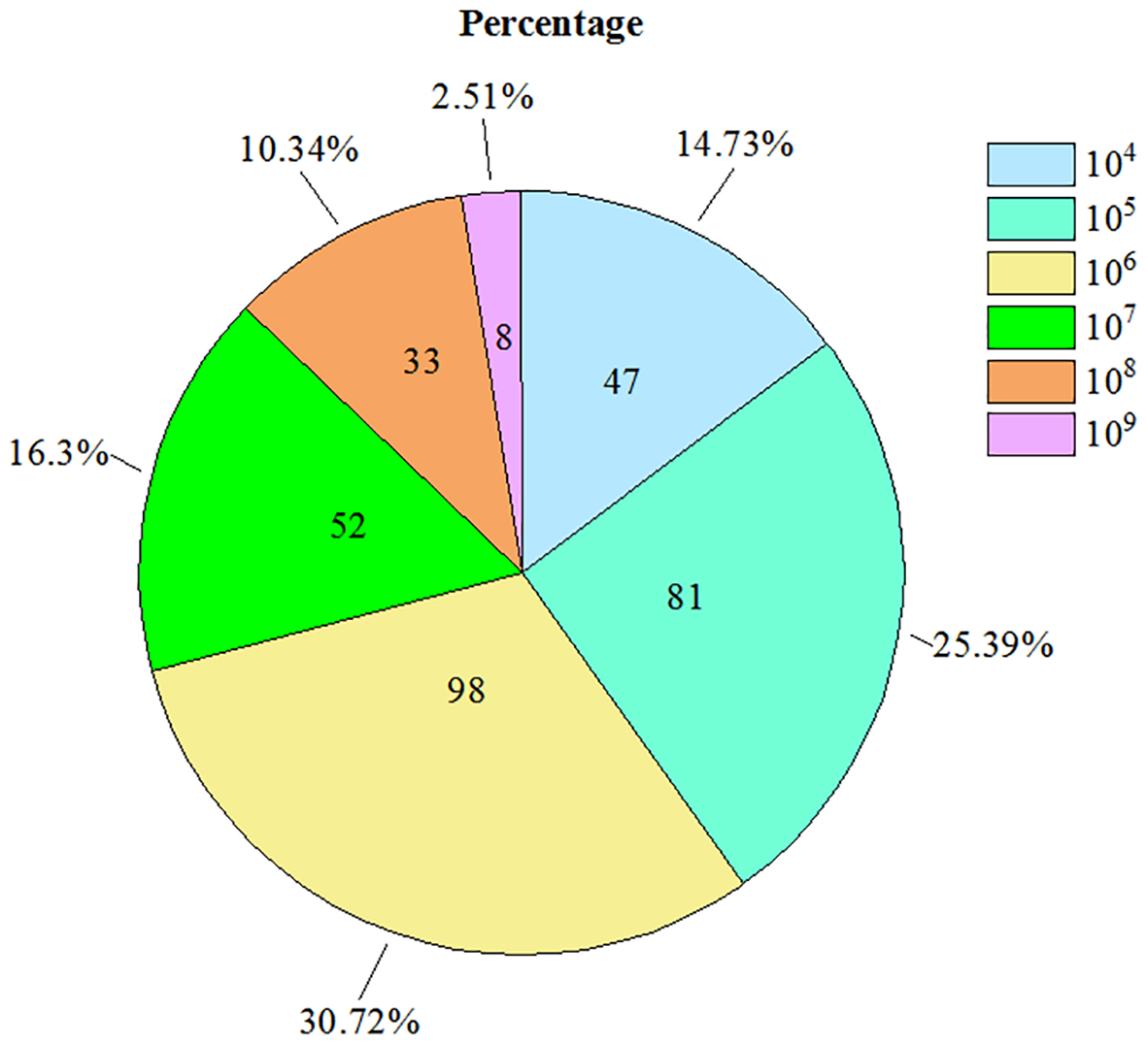
The detected amount of *Candidatus* Liberibacter asiaticus bacterium DNA by qPCR.

In total, 1037 insect samples were collected from different sites in the south of Zhejiang Province over a six year-period from 2014 to 2019. Of these samples, the presence of the bacterium was detected in 319 *D. citri* samples using RT-qPCR (**Table 1**). Copy numbers of the bacterium gene per nanogram of DNA ranged from approximately 10^4^ to 10^9^ (**Figure 2**), however, 10^4^–10^6^ copy numbers of the bacterium gene were detected in the majority of samples. Approximately 10^4^ copy numbers of the bacterium gene per nanogram of DNA were found in 47 samples, 10^5^ in 81 samples, and 10^6^ in 98 samples. The copy number of the bacterium gene per nanogram of DNA was 85.6% in 10^5^–10^6^. However, only eight samples were found with more than 10^9^ copy numbers of the bacterium gene per nanogram of DNA. These samples were collected from orchards located in the counties of Jiaojiang (2), Wenling (1), and Yuhuang (3) in 2014 and Wenling (2) in 2015. Although *D. citri* carrying bacteria were found in orchards in all counties, some *D. citri* samples lacked bacteria (**Table 1**).

### Proportion of *D. citri* carrying the *Ca.* L. asiaticus bacterium over a six-year period

**Figure 3:**
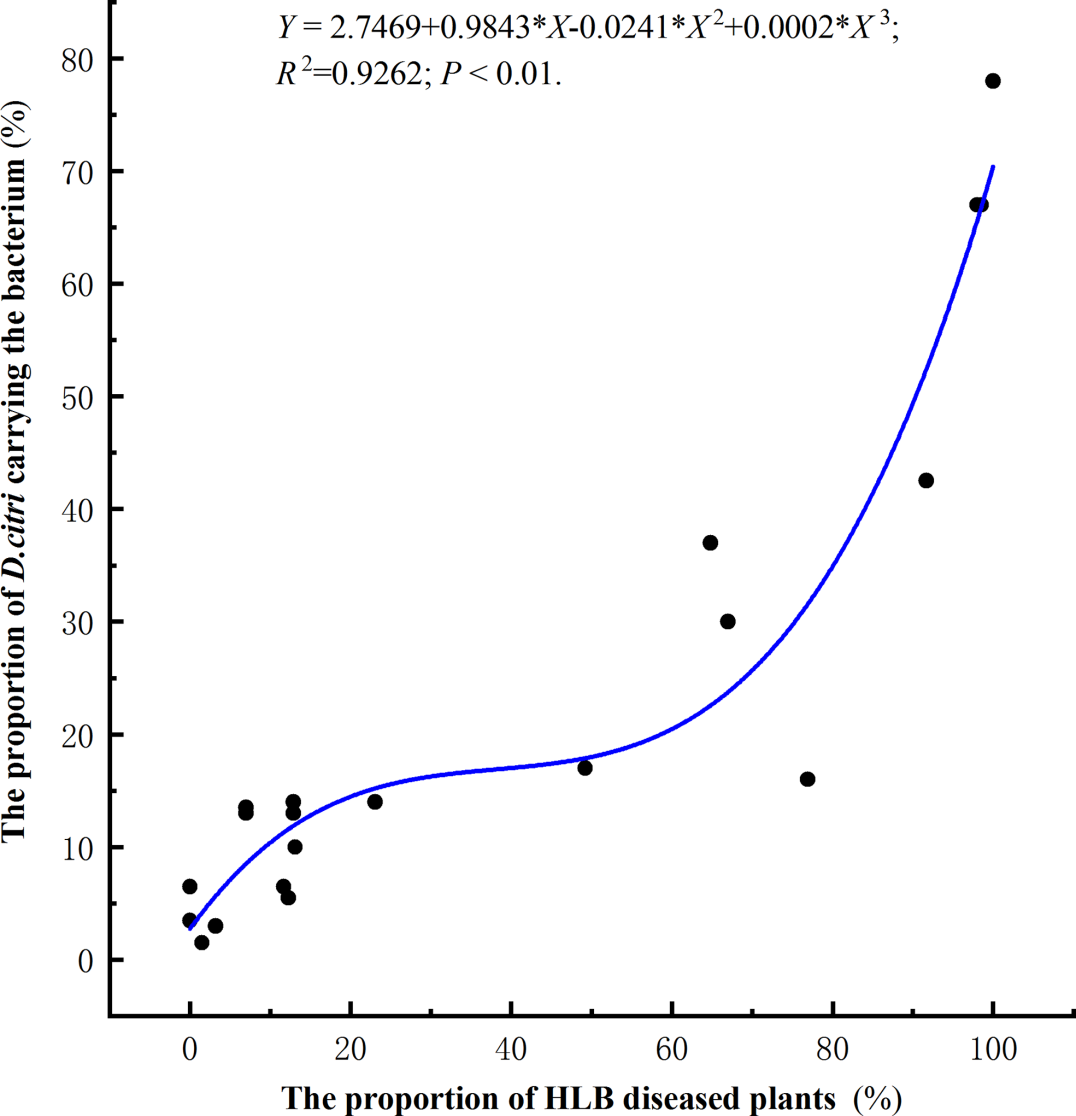
Proportion of *Diaphorina citri* carrying the pathogen *Candidatus* Liberibacter asiaticus between 2014 and 2019.

Among the 1037 samples, the proportion of *D. citri* carrying *Ca.* L. asiaticus decreased and finally stabilized over time. In 2014, *D. citri* were found at 220 sites and, *D. citri* were found to be carrying the bacterium in 92 sites accounting for 41.8% of these sites. In 2015, *D. citri* were found at 82 sites and *D. citri* were found to be carrying the bacterium in 27 sites accounting for 32.9% these sites. By 2016, 2017, 2018, and 2019, *D. citri* were found at 114, 144, 130, and 347 sites but *D. citri* were only found to be carrying the bacterium at 30.7% (35), 28.5% (41), 26.2% (34) and 25.9% (90) of these sites, respectively (**Figure 3**, **Table 1**).

### Changes in the proportion of *D. citri* carrying the bacterium throughout the year

**Figure 4:**
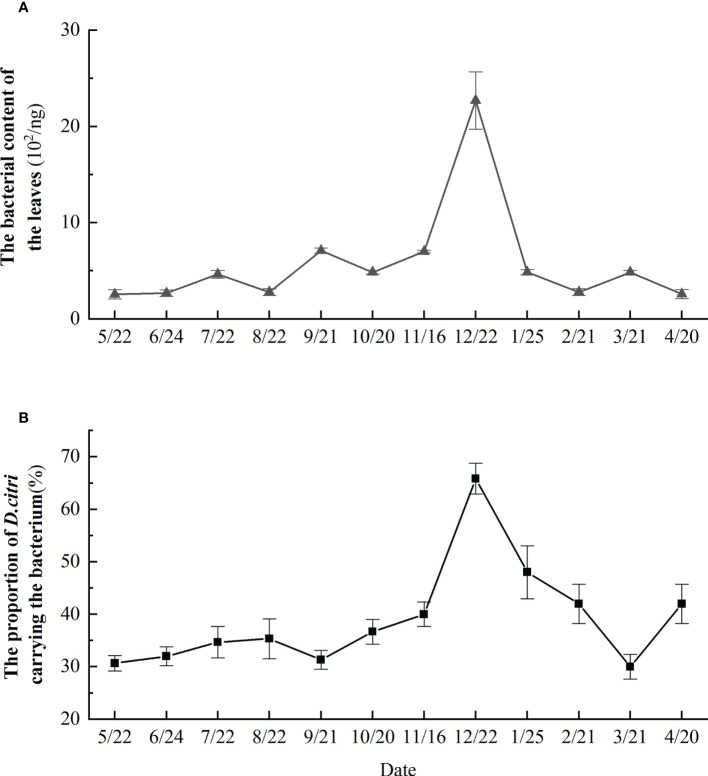
Proportion of. Monthly changes in *Candidatus* Liberibacter asiaticus bacterial content in citrus leaves over the course of a year **(A)**, and monthly changes in proportion of *Diaphorina citri* carrying the *Candidatus* Liberibacter asiaticus bacterium over the course of a year **(B)**.

Analysis of *D. citri* collected from a diseased orchards in Yueqing revealed that more than 30% of *D. citri* were carrying *Ca.* L. asiaticus throughout the year (**Figure 4B**). The proportion of *D. citri* carrying *Ca.* L. asiaticus gradually decreased from January to March and was stable and relatively low from May–September, then gradually increased and peaked in December (**Figure 4B**). The content of *Ca.* L. asiaticus in the citrus leaves was greatest in December. The content of *Ca.* L. asiaticus in the citrus leaves was relatively low in the fall and spring (close to 30% in May, June, September, and March). In spring and summer (February, April, May, June, and August), the bacterial content of citrus leaves was lowest, but reaching a high peak in December (**Figure 4A**).

### Relationship between the proportion of diseased plants and the proportion of *D. citri* carrying the bacterium

**Figure 5:**
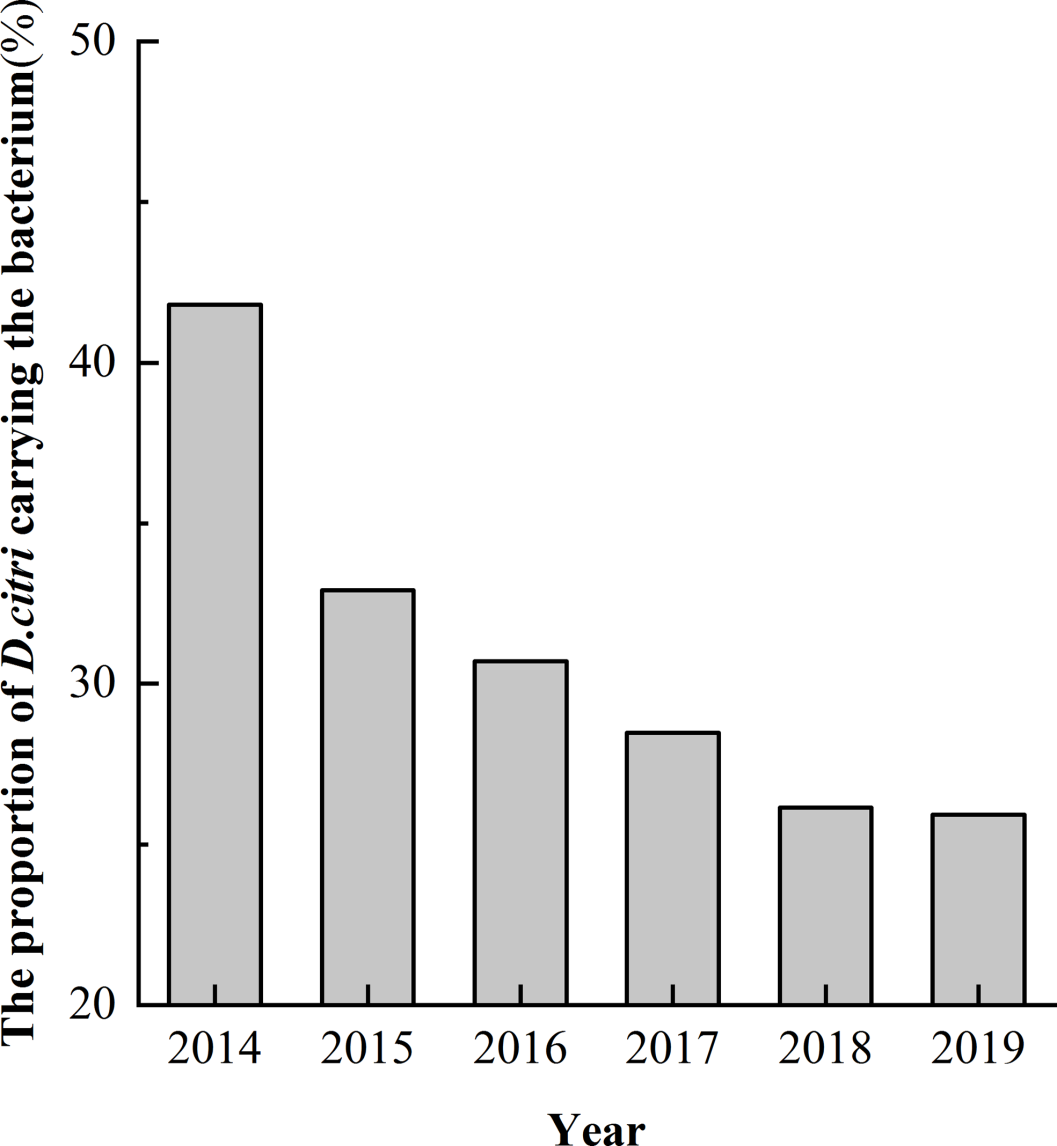
Correlation between the proportion of citrus plants with symptoms of HLB and the proportion of *Diaphorina citri* carrying the bacterium *Candidatus* Liberibacter asiaticus.

In total, 6600 *Citrus. unshiu* plants were investigated in 22 different orchards located in Huangyan, Yuhuan, Wenling, and Yueqing (**Table 2**). Based on investigations of 300 trees at each location, the proportion of diseased trees ranged from 0–98.5% (**Table 2**). Two hundred insects were collected at each location and were tested by RT-qPCR to determine the presence or absence of the bacterium. The proportion of diseased trees and the proportion of *D. citri* carrying the bacterium showed a significant positive correlation, and the number of plants showing symptoms of HLB increased as the proportion of *D. citri* carrying the bacterium increased (R^2^ = 0.93, P<0.01, **Figure 5**).

### The phyllosphere microbiomes differ between healthy and HLB citrus in April and December

**Figure 6:**
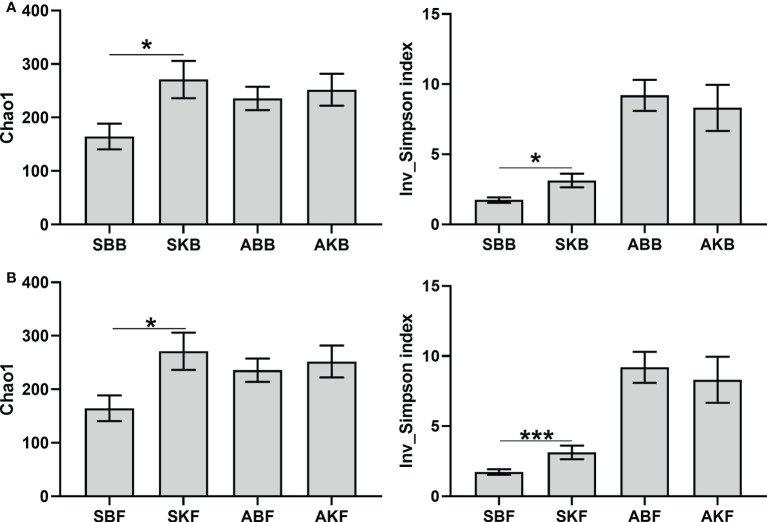
The α diversity indices of bacteria **(A)** and fungi **(B)** SBB, phyllosphere bacterial samples of spring disease leaves; SKB, phyllosphere bacterial samples of spring healthy leaves; ABB, phyllosphere bacterial samples of autumn disease leaves; AKB, phyllosphere bacterial samples of autumn healthy leaves; SBF, phyllosphere fungal samples of spring disease leaves; SKF, phyllosphere fungal samples of spring healthy leaves; ABF, phyllosphere fungal samples of autumn disease leaves; AKF, phyllosphere fungal samples of autumn healthy leaves. *P < 0.05, ***P < 0.001.

A total of 1626061 and 913399 high-quality sequences for bacteria and fungi were obtained after quality control of the original data using high-throughput sequencing technology. There were 17,590-184,257 for bacterial sequences and 27286-80816 for fungal sequences of each sample. Inv_Simpson index and Chao1 were used to evaluate α diversity of phyllosphere microorganisms with different treatments. The bacterial α diversity and the fungal α diversity in healthy leaves were significantly higher than that in diseased leaves in spring. But in autumn, there was no significant difference in α diversity of microbial communities between healthy and diseased citrus leaves (**Figure 6**).

**Figure 7:**
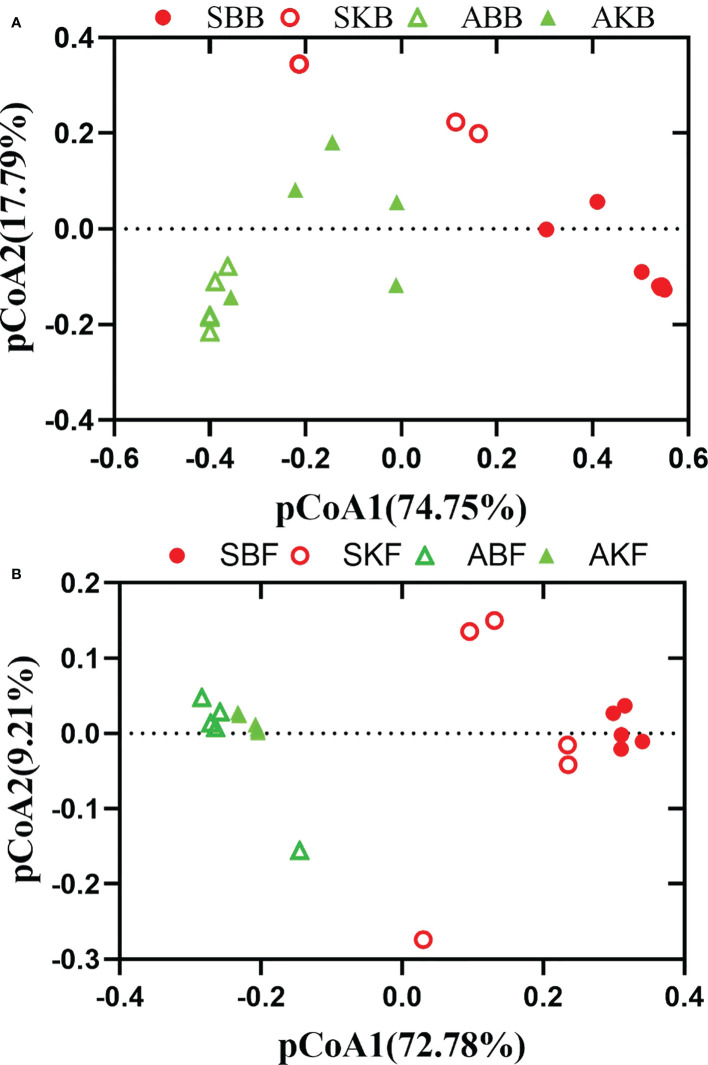
The pCoA of bacteria **(A)** and fungi **(B)** based on bray_cuits distance. SBB, phyllosphere bacterial samples of spring disease leaves; SKB, phyllosphere bacterial samples of spring healthy leaves; ABB, phyllosphere bacterial samples of autumn disease leaves; AKB, phyllosphere bacterial samples of autumn healthy leaves; SBF, phyllosphere fungal samples of spring disease leaves; SKF, phyllosphere fungal samples of spring healthy leaves; ABF, phyllosphere fungal samples of autumn disease leaves; AKF, phyllosphere fungal samples of autumn healthy leaves.

PERMANOVA and pCoA based on bray_cuits distance matrix were used to analyze phyllosphere microbial differences among different leaf group samples. The pCoA results showed that bacterial and fungal sampled in the phyllosphere healthy and diseased citrus leaves at the same time, could be completely separated (SBB and SKB, ABB and AKB). The phyllosphere bacterial and fungal samples in healthy and diseased citrus leaves at two time points were compared respectively, and there were also significant differences between SBB and ABB, and SKB and AKB groups (**Figure 7**). The results of dissimilarity analysis also showed that there was a significant difference in the phyllosphere microorganisms between healthy and diseased citrus leaves sampled in the same time and between healthy and diseased citrus leaves in different seasons (**Table S1**).

### The changes in bacteria and fungi OTUs by HLB pathogen invasion

**Figure 8:**
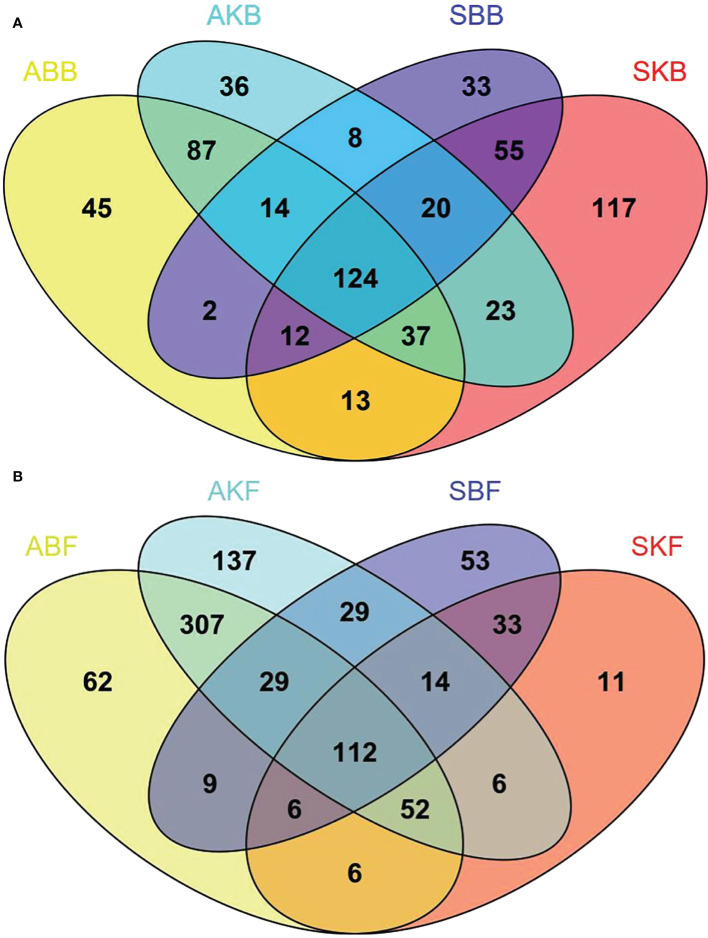
The analysis of united and shared OTU of bacteria **(A)** and fungi **(B)**. SBB, phyllosphere bacterial samples of spring disease leaves; SKB, phyllosphere bacterial samples of spring healthy leaves; ABB, phyllosphere bacterial samples of autumn disease leaves; AKB, phyllosphere bacterial samples of autumn healthy leaves; SBF, phyllosphere fungal samples of spring disease leaves; SKF, phyllosphere fungal samples of spring healthy leaves; ABF, phyllosphere fungal samples of autumn disease leaves; AKF, phyllosphere fungal samples of autumn healthy leaves.

In order to further study the phyllosphere microbial population structure of healthy and diseased leaf samples, the OTUs that were common and unique among these groups were analyzed and were plotted as a Venn plot (**Figure 8**). A total of 702 bacterial OTUs and 896 fungal OTUs were obtained. According to the results of Venn diagram, 124 (bacteria) and 109 (fungi) common OTUs were found in the phyllosphere samples of different treatments (**Figure 8A**). There were 269 common bacterial OTUs found in ABB group and AKB group, while 67 and 93 unique OTUs were found respectively in the two groups. And 206 common bacterial OTUs were found in SBB group and SKB group, while 65 and 193 unique OTUs were found in the two groups respectively. Besides, there were 163 and 515 common fungal OTUs found between ABF and AKF, SBF and SKF group (**Figure 8B**). 27 and 85 unique OTUs were found in ABF and AKF group, while 176 and 78 unique OTUs were found in SBB and SKB group, respectively.

## Discussion

Citrus HLB has become the most important quarantine disease in citrus-producing areas all over the world (Abdullah et al., 2009; Martinez et al., 2009; Bové, 2014; Puttamuk et al., 2014; da Graça et al., 2015). Once infected, the quite few of options to prevent the disease from spreading further, one of which is to cut off symptomatic branches. Since the development of the citrus industry and the scale of the expansion of citrus in world trade in recent years, clarifying the mechanism of the spread of *Ca.* L. asiaticus to find an effective control method for HLB is urgently required.

*Ca.* L. asiaticus has a long incubation period, therefore, infected citrus plants do not show symptoms of HLB during the early stages. In order to distinguish diseased plants from healthy plants, the RT-qPCR method is usually used to detect whether *Ca.* L. asiaticus bacteria are present (Fujikawa et al., 2013). In this study, *Ca.* L. asiaticus and the insect vector *D. citri* were detected from almost all southern citrus-producing regions of Zhejiang. This confirmed previous reports that HLB is an aggressive disease in citrus plantations and that it is widely distributed and spreading rapidly in Zhejiang Province (Zhou, 2020).

The dynamic of insect population is closely related to the growth rhythm and desirable food intake of insects, as well as the nutritional quality of host plants (Wallner, 1987). In addition, the phenological characteristics of host plants will also affect the growth of insects, leading to genetic variation among insect individuals and genetic differentiation among insect populations (Knolhoff and Heckel, 2014). Although tremendous progress has been made in understanding the ecological and evolutionary underpinnings of the Liberibacter disease pyramid, little is known about the quantitative relationship between these factors in the pyramid. By dynamically monitoring the bacterial content in the midvein of diseased leaves over the course of a year, we observed that values in December were more than 100 times higher than they were from March to May. This may be that citrus trees are cold-tolerant and evergreen, therefore, still lush in the fall, which significantly increasing the life span of *D. citri* (George et al., 2020). In addition, whether host plants can develop shoots in early spring and over the whole winter will affect the diapause of the insect population, which could exert a high selection pressure on the insect population to adapt to this extended host resource, and lead to species differentiation (Danks, 2013; Joyce et al., 2016). Furthermore, the chemoreceptors of insect are essential for the recognition and perception of plant secondary metabolites. Plant-eating insects can become conditioned to the host’s secondary metabolites and chemical constituents, which leads to change in insect behavior and drive host-associated differentiation (Powell et al., 2006; Medina et al., 2014). Moreover, the main rainy season in this area is from March to May. This long period of rain scours the bacterial communities on citrus trees, decreasing the number of bacteria on the leaves. From the beginning of July, the quantity of bacteria on leaves gradually increases. *Ca.* L. asiaticus can stand with high temperatures (Hoffman et al., 2013; Lopes et al., 2013). Our analyses showed that *D. citri* carried more bacteria in January and December than at other times, indicating that *D. citri* also has a certain level of cold tolerance. The *D. citri* life span is generally from spring to winter, by which time bacteria have fully proliferated in its body and, hence, the December insects were found to carry the most bacteria. In addition, large numbers of *D. citri with bacterium* were attracted to very few shoots in December. The abundance of *D. citri* on citrus shoots was highest in August and October, which is when their feeding and reproduction peaks. The rate of bacterial transmittance and population density also increased which may be related to the biotic factors that influence *D. citri* populations such as temperature and food. Although temperature is the main factor affecting the growth and development of insect, humidity has little effect on its survival and growth (Shang et al., 2013). *D. citri* carry a low level of bacteria in spring, which may be affected by climate and the bacterial content of the host tissue.

We found that the spread of HLB in Zhejiang Province was limited from 2014 to 2019. In 2014, *D. citri*-carrying bacteria were found at 41.8% of sites. By 2015, the level had dropped to 32.9% and by 2019 the level had dropped even further to 25.9%. This phenomenon may be related to the removal of diseased plants to control HLB. *D. citri* has a poor ability to fly long distances (Sakamaki, 2005). Long-range flight is made up of multiple short-range flights and relies on short-range wind diffusion. As a result, citrus HLB epidemics are dependent on sources of the pathogens and movement of the insect vectors. The removal of diseased plants therefore has a significant effect on controlling the spread of HLB (Abdullah et al., 2009; Bové, 2014).

Based on our observations, we showed a significant relationship between the plant infection rate and the proportion of *D. citri* carrying the bacterium (**Figure 5**). Our analysis showed that high levels of HLB disease in orchards correlate with high frequencies of *D. citri*-carrying bacteria. Coy and Stelinski (2015) found that infection levels of *Ca.* L. asiaticus in *D. citri* populations across Florida (USA) ranged from 37.5% to 100%, which was similar to the diverse infection levels found in different orchards in this study. Lee et al. (2015) have described a transmission mechanism that explains the high numbers of *Ca.* L. asiaticus-positive psyllids in retail environments based on an infection experiment. In retail environments, there are ample opportunities for each newly arriving the newly developed cluster of young leaves of healthy plant to be colonized by resident infected-psyllids, only after 15 days of being inoculated by *Ca.* L. asiaticus, the plants are infectious and transmit the pathogens to the next generation of psyllids. In this study of naturally infected orchards, we conclude that there is a relationship between the proportion of *D. citri* carrying the bacterium and the incidence of HLB disease. This correlation might due to that the physiological and biochemical characteristics of plants that have been infected with *Ca.* L. asiaticus may affect the behavior and performance of *D. citri* on susceptible plants. In addition, plant defense responses induced by the bacterial infection may indirectly affect *D. citri* by increasing the attractiveness of infected plants. *Ca.* L. asiaticus can also indirectly affect the adaptability of *D. citri*, which also affects the epidemic of citrus HLB. Host plant changes promote the genetic variation of insect adaptability and affects the genetic diversity and genetic structure of the insect population (Feder, 1995; Medina et al., 2014). *D. citri* infected with *Ca.* L. asiaticus bacteria spend more time feeding and show higher levels of fecundity than bacteria-free *D. citri* (Duan et al., 2009; Cen et al., 2012). *Ca.* L. asiaticus bacteria obtain nutrients and energy for their own invasion and proliferation by regulating the metabolic activities of substances in *D. citri* (Duan et al., 2009). These studies also indicated it must be correlation between the *D. citri* carrying with *Ca.* L. asiaticus and HLB disease incidence.

Controlling *D. citri* is the premise and foundation for preventing citrus HLB (Manjunath et al., 2008). At present, disease control strategies mainly involve chemical, physical, or biological methods of control. *D. citri* has a strong reproductive ability. Although chemical control has an obvious control effect on these insects (Boina et al., 2011), it is impossible to eliminate the insects completely, and chemical control can lead to resistance and enhance the reproductive ability of the insects. In addition, chemical control methods cause serious pollution and chemical residues can accumulate in the environment. As a result, the development of accurate and efficient pesticides to control *D. citri* has become a focus of research (Qureshi and Stansly, 2010). In this study, the proportion of the insect population carrying the bacterium changed over the course of the year, peaking in winter. Even though the insect population may be small, the application of pesticides could still be important to reduce the potential risk of *Ca.* L. asiaticus infection. Hybridization of resistant varieties using molecular biology and molecular breeding is another strategy for HLB control. No HLB symptoms were found in transgenic plants after grafting them with HLB-infected plants (Dutt et al., 2008). However, in the main citrus-producing industrial belt, controlling *D. citri* has been the main focus, and diseased plants have been destroyed by digging out and removing diseased trees (Wang and Trivedi, 2013), as well as high-density planting and shoot control to reduce *D. citri* feeding, strengthening field and orchard cultivation and management, and enhancing plant disease resistance, which can control citrus HLB to a great extent. Our observation of a reduction in disease levels in Zhejiang Province of China indicate that the application of these methods has had a significant effect on reducing the HLB disease epidemic. The phyllosphere (aboveground part of terrestrial plants) is an important niche of the plant, inhabited by diverse microbes which are collectively called the phyllosphere microbiome (Vorholt, 2012). The phyllosphere microorganisms could influence host plant by affecting nutrient acquisition, promoting host stress tolerance, altering plant hormones, and mediating plant pathogen interactions (Stone et al., 2018). The phyllosphere microbiomes were found to differ between infected and uninfected citrus leaves by melanose pathogen, and part of the phyllosphere microbiome shift could positively affect plant performance against pathogen invasion (Li et al., 2022).

Our analysis showed that there were significant differences among different healthy citrus leaves and HLB diseased leaves in April and December (**Figure 7**). The finding was consistent with Li et al. (2022), which also reported the changes of microbe community of citrus phyllosphere due to the invasion of fungal pathogen *Diaporthe citri*. HLB has been found to cause decreased relative abundance and/or expression activity of rhizoplane-enriched taxonomic and functional properties, hence collectively resulting in impaired plant host-microbiome interactions (Zhang et al., 2017). Our data showed that the effect of HLB may also apply on phyllosphere. It indicated that, in the early stage of citrus HLB, diversity of phyllosphere microorganisms is not only affected by the disease, but also regulated by seasonal factors. In spring, phyllosphere microorganisms showed a reverse trend of changes at bacteria and fungi in response to pathogenic invasion. The Venn results indicated that the OTUs number of diseased phyllosphere bacteria taxa were lower than Apirl citrus, but higher in December leaves phyllosphere fungal samples in both diseased and healthy leaves (**Figure 8**). The number of bacteria decreases, and fungi increases in the presence of pathogenic bacteria, which indicates that microbial communities exhibit ubiquitous taxa with special functions selected during pathogenic bacteria invasion.

## Conclusions

Through the long-term observation of citrus HLB, we conclude that there is a significant positive correlation between the level of citrus HLB in an orchard and proportion of *Ca.* L. asiaticus-carrying *D. citri*. The outbreak and spread of HLB in Zhejiang Province are declining. The number of bacteria in citrus leaves and the rate of *D. citri* carrying bacterium varies with seasonal factor. The phyllosphere bacterial and fungal communities were significantly different between healthy and disease phyllosphere in April and December. Pathogenic invasions change the citrus phyllosphere microbial community structure.

## Data availability statement

The datasets presented in this study can be found in online repositories. The names of the repository/repositories and accession number(s) can be found in the article/**Supplementary Material**.

## Author contributions

YM, EW, ZZ, and YH planted the experiment. YM, EW, MZ, and ZZ carry on the experiment. YW, LD, H-eZ, TT, WX, LL, XF, and LY analysis data. LY, LL, and YH write the manuscript. All authors contributed to the article and approved the submitted version.

## Funding

This study is funded by The National Key R & D Program of China (2021YFD1400800) and Key R & D Program of Hunan Province, China (2022NK2052).

## Conflict of interest

The authors declare that the research was conducted in the absence of any commercial or financial relationships that could be construed as a potential conflict of interest.

## Publisher’s note

All claims expressed in this article are solely those of the authors and do not necessarily represent those of their affiliated organizations, or those of the publisher, the editors and the reviewers. Any product that may be evaluated in this article, or claim that may be made by its manufacturer, is not guaranteed or endorsed by the publisher.
