# Supplementary material for: Citrus Huanglongbing correlated with incidence of Diaphorina citri carrying Candidatus Liberibacter asiaticus and citrus phyllosphere microbiome
Source: Front Plant Sci. 2022 Nov 18;13:964193. doi: 10.3389/fpls.2022.964193 (PMC9716883; doi:10.3389/fpls.2022.964193)
Supplement: Supplementary file 1 [file DataSheet_1.docx]

**Table S1 Dissimilarity test (PERMANOVA) of bacterial and fungal communities between healthy and disease citrus phyllosphere samples.**

| **Taxonomy** | **Group** | **PERMANOVA** | |
| --- | --- | --- | --- |
|  |  | **F** | **p** |
|  | **SBB-SKB** | 5.9305 | 0.037 |
| **Bacteria** | **SBB-ABB** | 48.1609 | 0.006 |
|  | **SKB-AKB** | 2.8865 | 0.018 |
|  | **ABB-AKB** | 3.9003 | 0.035 |
| **Fungi** | **SBF-SKF** | 4.3547 | 0.008 |
|  | **SBF-ABF** | 31.2775 | 0.008 |
|  | **SKF-AKF** | 13.4364 | 0.009 |
|  | **AKF-ABF** | 2.3867 | 0.009 |

**Table S2 The properties of the empirical and random networks among healthy and disease citrus phyllosphere samples.** KB: healthy and disease citrus phyllosphere bacterial and fungal samples.

| **Groups** | **Empirical Network** | | | | | | **Random networks(100)** | | |
| --- | --- | --- | --- | --- | --- | --- | --- | --- | --- |
|  | **RMT threshold** | **nodes** | **links** | **Average clustering coefficient**  **(avgCC)** | **Average path distance (GD)** | **Modularity**  **(fast_greedy)** | **Average clustering coefficient**  **(avgCC)** | **Average path distance**  **(GD)** | **Modularity**  **(fast_greedy)** |
|  |  |  |  |  |  |  |  |  |  |
| KB | 0.96 | 64 | 242 | 0.035 | 2.62 | 0.2 | 0.292 +/- 0.033 | 2.351 +/- 0.057 | 0.218 +/- 0.010 |

**Figure S1 The OTU that relative abundance was significantly higher or lower than healthy citrus phyllosphere samples.**

**
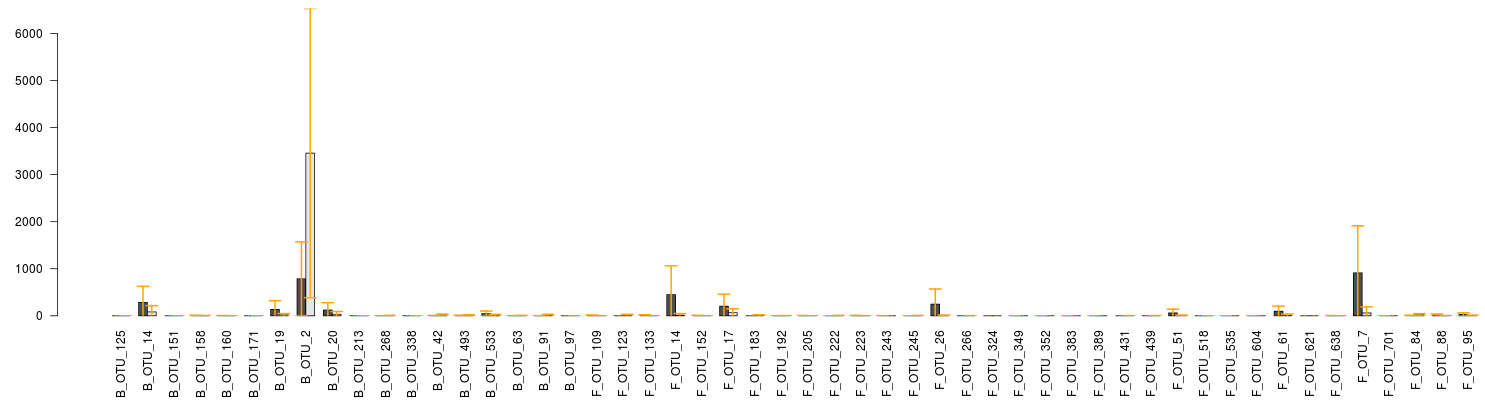
**
